# Association Between Suicidal Ideation and Negative Affect: 6-Month Ecological Momentary Assessment Study

**DOI:** 10.2196/85073

**Published:** 2026-09-23

**Authors:** Alejandro Porras-Segovia, Ana Pérez-Balaguer, Adrián Alacreu-Crespo, Maria Luisa Barrigón, Paula García-Barja, Philippe Courtet, Jorge López-Castroman, Inmaculada Peñuelas-Calvo, Enrique Baca-García

**Affiliations:** 1 Health Research Institute Fundación Jiménez Díaz, Madrid, Spain; 2 Department of Psychiatry, University Hospital Rey Juan Carlos, Móstoles, Madrid, Spain; 3 Department of Psychiatry, University Hospital El Escorial, San Lorenzo de El Escorial, Madrid, Spain; 4 Department of Psychology and Sociology, University of Zaragoza, Zaragoza, Spain; 5 Institute of Psychiatry and Mental Health, Hospital General Universitario Gregorio Marañón, IiSGM, CIBERSAM, ISCIII, School of Medicine, Universidad Complutense, Madrid, Spain; 6 IGF, Univ. Montpellier, CNRS, INSERM, Montpellier, France; 7 Department of Emergency Psychiatry and Acute Care, Lapeyronie Hospital, CHU Montpellier, Montpellier, France; 8 Department of Psychiatry, Nimes University Hospital, Nimes, France; 9 CIBERSAM, research group CB/07/09/0025, Madrid, Spain; 10 Department of Psychiatry, Radiology, Public Health, Nursing and Medicine, University of Santiago de Compostela, Santiago de Compostela, Spain; 11 Department of Child and Adolescent Psychiatry, University Hospital 12 de Octubre, Madrid, Spain; 12 Department of Psychiatry, Universidad Complutense de Madrid, Madrid, Spain; 13 Health Research Institute Hospital 12 de Octubre (i+12Institute), Madrid, Spain; 14 CIBERSAM-ISCIII (Biomedical Research Networking Centre for Mental Health), Madrid, Spain; 15 Department of Psychiatry, Hospital Universitario Fundación Jiménez Díaz, Avda. de los Reyes Católicos, 2, Madrid, 28040, Spain, 34 91 541 72 67, 34 91 542 35 36; 16 CIBERSAM, research group CB/07/09/0025, Av. Monforte de Lemos 3-5. Pabellon 11. Planta 0, Madrid, Spain, 34 91 822 20 73; 17 Department of Psychiatry, Universidad Autónoma de Madrid, Calle Arzobispo Morcillo, 4, Madrid, Spain, 34 91 497 5448; 18 Department of Psychiatry, Hospital Universitario General de Villalba, Collado Villalba, M-608 Carretera, M-608, Km 41, Madrid, Spain, 34 910 90 81 02; 19 Department of Psychiatry, Hospital Universitario Infanta Elena, Valdemoro, Av. de los Reyes Católicos, 21, Madrid, Spain, 34 918 94 84 10

**Keywords:** suicide ideation, suicide attempt, ecological momentary assessment, eHealth, mHealth, mobile health, new technologies

## Abstract

**Background:**

Suicide is a major public health challenge. Traditional assessments of suicidal thoughts and behaviors rely on retrospective measures that are subject to recall bias and show limited predictive accuracy. Ecological momentary assessment (EMA) can improve suicide risk assessment by capturing real-time information in participants’ natural environments. However, EMA burden often results in short follow-up periods.

**Objective:**

This study aimed to examine the association between suicidal ideation (SI) and multiple variables collected in real time using smartphone-based EMA over 6 months, with the goal of identifying short-term predictors of suicide risk in a clinical population. A secondary objective was to explore whether baseline depression and psychological pain predicted EMA-reported SI.

**Methods:**

This is a prospective EMA-based study carried out among outpatients at high risk of suicide, recruited in Madrid, Spain. At baseline, depression, suicidality, and psychological and physical pain were assessed using traditional questionnaires (Inventory of Depressive Symptomatology, Columbia Suicide Severity Rating Scale, and Visual Analog Scales). During the 6-month follow-up, participants completed daily smartphone-based EMA prompts assessing SI, nonsuicidal self-injury, negative affect, interpersonal experiences, sleep, and eating habits. Each day, 2-4 questions derived from validated instruments were randomly selected from a pool of 34 items to minimize participant burden. Mixed-effects models examined within-person variability, temporal trends, and real-time associations between EMA variables and SI. Intraindividual variability was quantified using intraclass correlation coefficients and root mean squared successive differences. Linear regression analyses explored associations between EMA variables and demographic covariates and evaluated whether baseline depression and psychological pain predicted the mean and variability of SI and negative affect.

**Results:**

A total of 99 participants were included in the final analysis, and 13,900 EMA responses were obtained. The slope of SI showed no significant changes over time, with similar results for negative affect, interpersonal difficulties, sleep problems, and appetite. Substantial within-person variability was observed across all EMA domains. Negative affect (ß=0.193, SE 0.03; *t*_98_=6.21; *P*<.001) and interpersonal difficulties (ß=0.03, SE 0.168; *t*_98_=5.96; *P*=.001) were significantly associated with SI and remained significant in combined models. In a subsample of 37 participants, lower baseline psychological pain was associated with lower levels of negative affect and SI over time, whereas higher baseline depression was associated with greater negative affect.

**Conclusions:**

This study provides novel insights into the real-time relationship between SI and key psychological and behavioral variables, particularly negative affect and interpersonal difficulties, using smartphone-based EMA in a high-risk clinical population. The substantial within-person variability highlights the dynamic nature of suicide risk and the limitations of traditional assessments. Future studies should aim to translate EMA-based monitoring into actionable interventions to provide real-time support for individuals at high risk of suicide.

## Introduction

Suicide is a significant global public health concern, accounting for over 700,000 deaths worldwide each year [1]. Years of potential life lost due to suicide have declined less than those associated with other external causes and have even increased in some regions [2]. For example, in 2020, suicide accounted for almost as many years of potential life lost as the novel coronavirus (SARS-CoV-2) in the United States [3] and even surpassed it in Spain [4]. Nevertheless, suicide continues to receive far less public health attention than physical illnesses.

Traditionally, suicidal thoughts and behaviors (STBs) have been assessed through clinical interviews and self-report questionnaires. However, these methods face notable limitations, including limited predictive accuracy, insufficient consideration of contextual influences, and susceptibility to recall bias [5]. Furthermore, much of the research has emphasized long-term rather than immediate (proximal) risk factors for STB [6].

Ecological momentary assessment (EMA) has emerged as a promising tool to address some of these limitations. EMA involves repeatedly assessing participants in real time, typically through daily prompts delivered in their natural environments [7]. While EMA was originally administered via paper diaries, it is now predominantly conducted through smartphones [8]. This approach reduces recall bias, enables tracking of dynamic changes over time, and captures subtle variations that traditional methods often overlook [9].

Systematic reviews have highlighted the potential of EMA for real-time monitoring of STB [8,10]. Studies have demonstrated the feasibility and acceptability of EMA across various populations [11,12] and its potential for examining psychological mechanisms. For example, Herzog et al [13] identified a correlation between suicidal ideation (SI) and deficits in attentional control. Other studies suggest that previous SI and shifts in emotional or cognitive states may predict SI the following day [14]. Furthermore, individuals who report SI demonstrate higher levels of perceived burdensomeness and a lower sense of belonging [15], while feelings of entrapment appear to be associated with short-term SI in both directions [16]. However, challenges remain, particularly with regard to the burden of EMA, which refers to participant fatigue due to frequent prompts. This can reduce adherence and limit the duration of data collection to just a few weeks [8,17].

In recent years, our research group has sought to mitigate some of these limitations through the SmartCrisis 1.0 project [18]. By implementing a rotating system of questions to reduce repetition, we achieved a higher level of feasibility than in previous EMA studies conducted in real-world clinical settings, even in the absence of financial incentives [19,20].

However, SmartCrisis 1.0 had some significant limitations. For example, it focused solely on passive SI, which restricted the scope and applicability of its findings. To address this issue, we developed SmartCrisis 2.0—an enhanced version that incorporates active suicide intervention measures and integrates a safety plan for managing suicide risk. Initial results suggest high user satisfaction, as evidenced by strong ratings in user surveys [21].

The current study aims to investigate the association between STB and various variables measured in real time via smartphone-based EMA, with the goal of identifying short-term predictors of suicide risk in a clinical population. A secondary aim is to examine how baseline levels of depression and psychological distress influence variability in EMA-reported SI.

## Methods

### Setting and Design

This prospective study used EMA and focused on psychiatric outpatients who were identified as being at high risk of suicide. Participants were recruited from 4 hospitals in the Community of Madrid (Spain). This study is part of the SmartCrisis 2.0 project, a randomized controlled trial (RCT) with a parallel-group design. The trial includes patients with a recent history of suicidal behavior, who are randomly assigned to 1 of 2 groups. Participants in the control group receive treatment as usual together with EMA, whereas participants in the intervention group receive treatment as usual plus EMA and an ecological momentary intervention (EMI). Both groups are followed for up to 12 months, with repeated clinical assessments and continuous smartphone-based monitoring. EMA data is collected continuously throughout the follow-up period in both groups; therefore, for participants in the intervention arm, EMA monitoring occurs concurrently with the EMI, whereas in the control group, EMA is conducted without intervention. The EMI is a smartphone-based intervention that includes a personalized safety plan, an enhanced contact system with supportive messages, and a toolbox of brief therapeutic exercises delivered via the app. Full details of the SmartCrisis 2.0 RCT protocol are available in a separate publication [7]. Although the broader SmartCrisis 2.0 project includes an interventional element, the present study focuses on the observational EMA monitoring data collected during the study period and does not evaluate the effectiveness of the intervention.

The original protocol planned to recruit 220 participants across 5 sites, including centers in France. However, the French sites were ultimately withdrawn, and the study proceeded with the 4 Spanish sites included in the present study. In addition, although the target sample size for the RCT is 220 participants, the present analysis includes a smaller sample because sufficient statistical power had already been achieved to address the aims of this study.

### Ethical Considerations

The study adhered to the ethical principles set forth in the Declaration of Helsinki and was approved by the Research Ethics Committee of Fundación Jiménez Díaz (EC005-21_FJD). Written informed consent was obtained from all participants. To ensure confidentiality, the usernames and personal information of participants were pseudonymized using coded identifiers. All data were encrypted using AES-256 with 256-bit keys and were managed within a professional key management system, which underwent external security compliance audits. No financial compensation was offered to participants.

### Terminology and Definitions

In this study, STBs are used as an umbrella term encompassing different suicide-related experiences, including:

1. SI: a cognitive process involving thoughts about the possibility of ending one’s life. This includes both passive wishes to be dead and active thoughts of killing oneself, in the absence of associated preparatory behavior.
2. Suicide attempt: a self-directed injurious behavior accompanied by intent to die and involving a real risk to life, regardless of the outcome.
3. Nonsuicidal self-injury: a deliberate self-injurious behavior without intent to die, typically aimed at relieving emotional distress, regulating affect, or causing changes in the external environment.
4. Death by suicide: a fatal self-directed injurious act with intent to die.

Definitions were based on the Columbia–Suicide Severity Rating Scale [22].

Negative affect refers to a broad dimension of emotional distress that includes unpleasant emotional states such as anger, guilt, fear, worry, shame, and nervousness, as originally described by Watson et al [23]. In the present study, this construct was operationalized using EMA items assessing stress, restlessness, hopelessness, anxiety, sadness, psychological pain, and anger directed toward oneself or others, along with a positively worded happiness item that was coded inversely.

### Sample

The study sample comprised psychiatric outpatients who had recently experienced STBs. The inclusion and exclusion criteria are listed in Textbox 1.

For the final analysis, only those participants who responded to at least 3 EMA prompts related to STB were included.

Textbox 1. **Inclusion and exclusion criteria.**

Inclusion criteria

- Age 18 years or older
- A suicide attempt or emergency intervention for suicidal intention (SI) within the previous month
- Capacity to understand the study and provide informed consent
- Fluency in Spanish
- Access to a personal smartphone with internet connectivity

Exclusion criteria

- Refusal to install the mobile app
- Inability to understand or sign the informed consent
- No regular access to a compatible smartphone

### Measures and Procedure

#### Recruitment

Psychiatrists conducting routine clinical care were responsible for recruitment. They evaluated patients for eligibility, explained the study objectives, and invited those who met the criteria to participate. Those who agreed were asked to sign a written informed consent form. No financial incentives were provided for participation.

#### Clinical (Face-to-Face) Measures

A trained psychologist conducted a baseline clinical interview, during which demographic and clinical data were collected. Standardized instruments were used to assess the following dimensions:

- Depression: Inventory of Depressive Symptomatology–Clinician Rated (IDS-C). The IDS-C has demonstrated good internal consistency, high interrater reliability, and strong concurrent validity [24].
- STBs: Columbia–Suicide Severity Rating Scale. The Columbia–Suicide Severity Rating Scale has demonstrated good validity, sensitivity to clinical change, and predictive validity for near-term suicidal behavior [22].
- Psychological and physical pain: Visual analog scales. Visual analog scales have demonstrated strong convergent validity, sensitivity to changes in pain severity, and good feasibility among individuals in clinical populations [25].

Data on psychological pain were obtained later in the study being available only for a subsample of participants. Additional demographic details, including date of birth and gender identity, were obtained from electronic health records.

#### EMA Measures

During the baseline interview, the MEmind mobile app was installed on participants’ smartphones. Participants were shown how to use the app, and any questions were answered; no additional training was required. This app delivers brief, daily surveys directly to the device. Each day, at a random time between 9:00 AM and 9:00 PM, a prompt containing 2-4 questions (4 in the first 2 months, and 2 thereafter) was presented. Participants could complete the questionnaire until midnight of the same day. At least 1 item related to SI was included in each daily questionnaire, while the remaining questions were randomly selected from a pool of 34 items based on validated instruments, including

- Salzburg Suicide Process Questionnaire [26]
- Patient Health Questionnaire-9 [27]
- Positive and Negative Affect Schedule [23]
- Interpersonal Needs Questionnaire [28]
- Prior EMA research on suicidality

These instruments have established evidence of reliability and validity. Specifically, the Patient Health Questionnaire-9, the Positive and Negative Affect Schedule, and the Interpersonal Needs Questionnaire have demonstrated good internal consistency, as well as construct and concurrent validity [23,27,28]. Although the formal psychometric properties of the Salzburg Suicide Process Questionnaire have not yet been reported, its items were selected from previously validated instruments and adapted for real-time monitoring of suicidal processes [26].

The EMA questions were grouped into the following categories (Multimedia Appendix 1):

- SI: 5 items
- Nonsuicidal self-injury: 2 items
- Negative affect: 9 items
- Interpersonal experiences: 11 items
- Sleep: 4 items
- Eating habits: 3 items

This random and varied delivery of items was specifically designed to minimize the burden typically associated with EMA, which often leads to fatigue among participants and high dropout rates. While this approach reduces redundancy, it requires questions to be grouped into broader categories for statistical analysis. Items that were positively worded (eg, “I feel full of hope”) were reverse-coded to align with their category.

Participants were observed over a 6-month period.

### Statistical Analysis

All statistical analyses were performed using the R software package (R Foundation for Statistical Computing). The intraindividual mean and variability of responses were calculated for each participant. Variability was assessed using the root mean square of successive differences. Missing data were not imputed; mixed models were chosen for statistical analysis as they are robust in dealing with missing values [29].

Mixed-effects models were used to evaluate within-person variability and temporal trends. Between-person reliability (R-between), within-person reliability (R-within), and intraclass correlation coefficients (ICCs) were calculated from the null model to quantify variability [30]. Reliability coefficients were interpreted according to Shrout (1998) as follows [31]: 0.00‐0.10 indicates no reliability, 0.11‐0.40 slight reliability, 0.41‐0.60 fair reliability, 0.61‐0.80 moderate reliability, and 0.81‐1.00 substantial reliability. Time was then entered as a fixed effect, participants as random effects, and each EMA category (eg, negative affect or sleep) as the dependent variable.

Mixed-effects models were also used to examine the effect of EMA categories (eg, negative affect, interpersonal difficulties, sleep, and eating behavior) on SI over time. Each model included fixed effects for the EMA category, time, and their interaction. Random intercepts were specified for participants, with random slopes for time. An additional model was run that included all significant EMA categories together.

Multiple linear regression analyses were conducted to assess the association between EMA variables and demographic covariates (age and gender). Further regressions examined whether baseline levels of depression and psychological distress predicted the mean and variability of SI and negative affect throughout the EMA follow-up period. Where appropriate, posthoc simple slope analyses were performed.

All tests were 2-tailed and significance was set at *P*<.05, with 95% CIs.

## Results

### Characteristics of the Sample

A total of 115 participants were recruited. Of the initial sample, 99 had sufficient data (at least 3 EMA answers) and were included in the final analysis. The mean age was 39.5 (SD 0.33) years (43.4, SD 2.24 y for men and 37.1, SD 1.74 y for women), and there was a majority of female participants (n=62, 62.6% females vs n=37, 37.4% males).

Most participants had completed secondary education (n=71, 72.1%), followed by those with university education (n=25, 25%), while a small proportion had not completed compulsory education (n=3, 2.9%). Regarding marital status, 49% (n=49) of participants were single, 17.1% (n=17) were separated, divorced, or widowed, and 33.9% (n=34) were in a relationship. Additionally, 31.3% (n=31) of the sample consisted of individuals who were employed or studying.

There were 87.9% (n=87) participants with a lifetime mood disorder, 67.7% (n=67) participants with a lifetime anxiety disorder, 63.6% (n=63) participants with a lifetime psychotic disorder, 43.4% (n=43) participants had borderline personality disorder, and 22.2% (n=22) participants had a history of substance or alcohol use disorders.

### Description of EMA Variables

A total of 13,900 EMA responses were obtained, corresponding to an approximate overall response rate of 29.3%. Of these, 5638 belonged to the SI category. The slope of SI over time was not statistically significant (β=−0.006, SE 0.014; *P*=.68), showing no significant changes over follow-up. Similar results were obtained for the slopes of negative affect (β=−0.019, SE 0.017; *P*=.24), interpersonal difficulties (β=−0.027, SE 0.019; *P*=.15), sleep problems (β=0.007, SE 0.011; *P*=.52), and appetite (β=0.001, SE 0.018; *P*=.99).

SI had an R-between of 0.51, an R-within of 0.84, and an ICC of 0.17. Negative affect showed an R-between of 0.74, an R-within of 0.83, and an ICC of 0.24. Interpersonal problems had an R-between of 0.79, an R-within of 0.84, and an ICC of 0.26. Sleep problems showed an R-between of 0.63, an R-within of 0.77, and an ICC of 0.29. Finally, appetite showed an *R*-between of 0.53, an *R*-within of 0.62, and an ICC of 0.45. Between-person reliability ranged from fair to moderate, while within-person reliability was good to high across constructs. ICCs were generally low across constructs, except for appetite.

Of the total sample, 56% (n=55) of participants received the safety plan intervention during EMA monitoring. We tested whether the intervention had an effect on SI outcomes. The effect of the safety plan was not significant for SI (β=−1.24; *t*_98_=−0.47; *P*=.64), nor for the intraindividual mean (β=−.32; *t*_98_=−0.11; *P*=.91) or variability (β=−1.29; *t*_98_=−0.54; *P*=.59) of SI. For this reason, the safety plan intervention was not included as a covariate in the remaining models.

**Figure 1.**
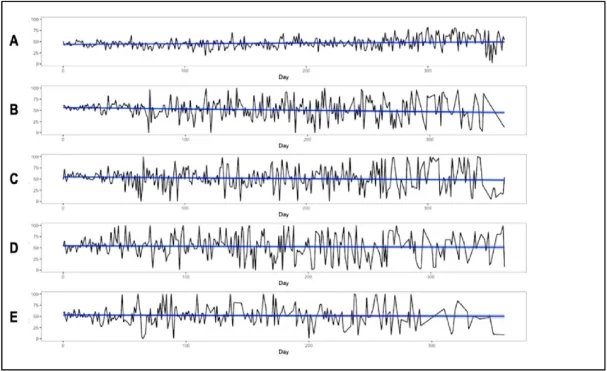
Fluctuation of ecological momentary assessment variables over time. (A) Suicidal ideation. (B) Negative affect. (C) Interpersonal experiences. (D) Sleep. (E) Eating habits.

Figure 1 illustrates the evolution of the EMA responses over time.

### Association Between EMA Variables and SI

The variables statistically significantly associated with SI were negative affect (β=0.193, SE 0.03; *t*_98_=6.21; *P*<.001) and interpersonal difficulties (β=0.17, SE 0.03; *t*_98_=5.96; *P*<.001). The association was positive: the greater the negative affect and the interpersonal difficulties, the greater the SI. When negative affect and interpersonal difficulties were put together in the same model, both predictors maintained their significance (*P*<.001).

The effect of the interactions negative affect × time (*P*=.12) and interpersonal difficulties × time (*P*=.29) were not statistically significant. There were also no statistically significant effects on appetite (appetite, *P*=.54; appetite × time, *P*=.78) and sleep models (sleep, *P*=.97; sleep × time, *P*=.15).

Greater intraindividual means of negative affect (*R*^2^=0.39; β=0.502, SE 0.08; *t*_98_=6.65; *P*<.001) and interpersonal difficulties (*R*^2^=0.182; β=0.359, SE 0.07; *t*_98_=5.03; *P*<.001) were associated with greater intraindividual means of SI. No significant differences were found for sleep problems and appetite. None of the intraindividual variability indexes were associated with the intraindividual mean of SI.

Intraindividual variability of SI was directly associated with intraindividual variability in the rest of the EMA variables: the greater the SI variability, the greater the variability of negative affect (*R*^2^=0.15; β=0.477, SE 0.12; *t*_98_=4.04; *P*<.001), interpersonal difficulties (*R*^2^=0.15; β=0.323, SE 0.07; *t*_98_=4.49; *P*<.001), sleep problems (*R*^2^=0.12; β=0.259, SE 0.09; *t*_98_=2.96; *P*=.004), and eating habits (*R*^2^=0.23; β=0.318, SE 0.09; *t*_98_=3.54; *P*<.001).

### Baseline Psychological Pain as a Predictor of Intraindividual Mean and Variability

A subsample of 37 (48.6% females, mean age 40.6, SD 2.41 y) participants had information about baseline psychological pain and depression. The mean baseline IDS-C score of the participants was 26.2 (SD 1.59). Their current and maximum psychological pain at inclusion was 6.76 (SD 0.48) and 7.36 (SD 0.59), respectively. Participants answered EMA questions for a mean of 146 (SD 21.2) days.

**Figure 2.**
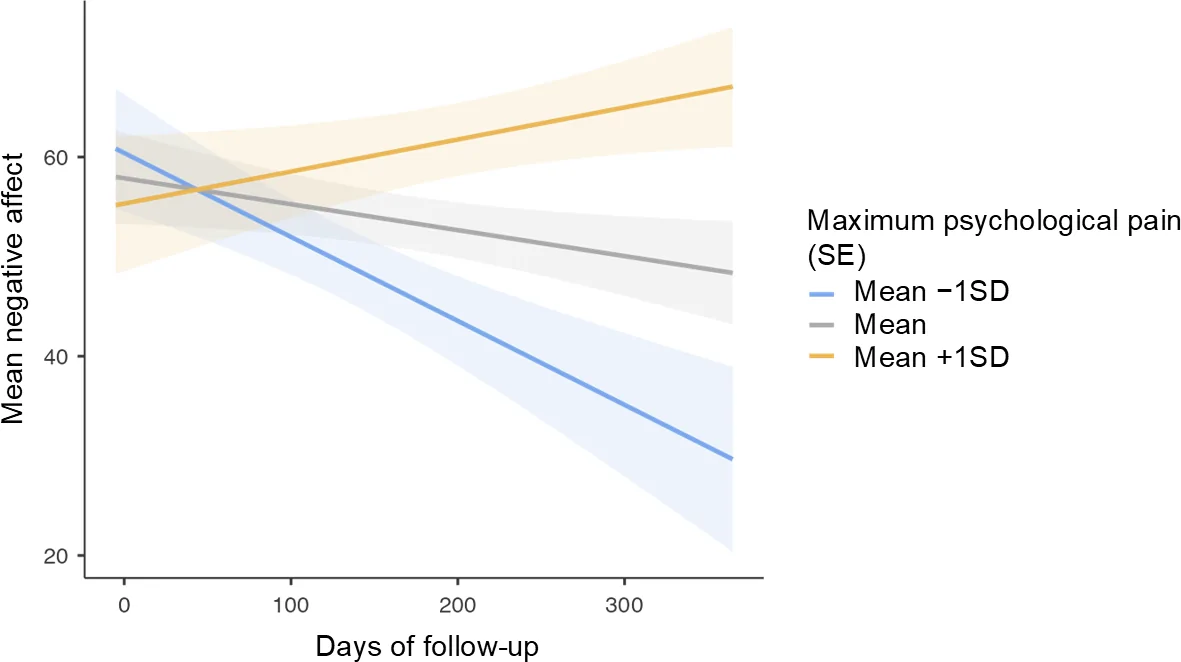
Association between baseline psychological pain and ecological momentary assessment–measured negative affect.

There was a negative association between baseline maximum psychological pain and variability of negative affect (*R*^2^=0.12; β=−1.40, SE 0.65; *t*_98_=−2.16; *P*=.04) but psychological pain itself did not predict the mean of negative affect (*P*=.41). Thus, the greater the psychological pain, the lower the variability. The interaction psychological pain × days of follow-up significantly predicted intraindividual negative affect mean (*R*^2^=0.24; β=0.019, SE 0.007; *t*_98_=2.58; *P*=.02). Simple slopes showed significant results for the –1SD psychological pain (β=−0.08, SE 0.037; *P*=.03) but not for the +1 SD of psychological pain (β=0.04, SE 0.039; *P*=.19); showing patients with lower psychological pain and longer follow-up experiments less negative affect (Figure 2). The interaction psychological pain × days of follow-up did not predict the intraindividual variability of negative affect (*P=.*21).

**Figure 3.**
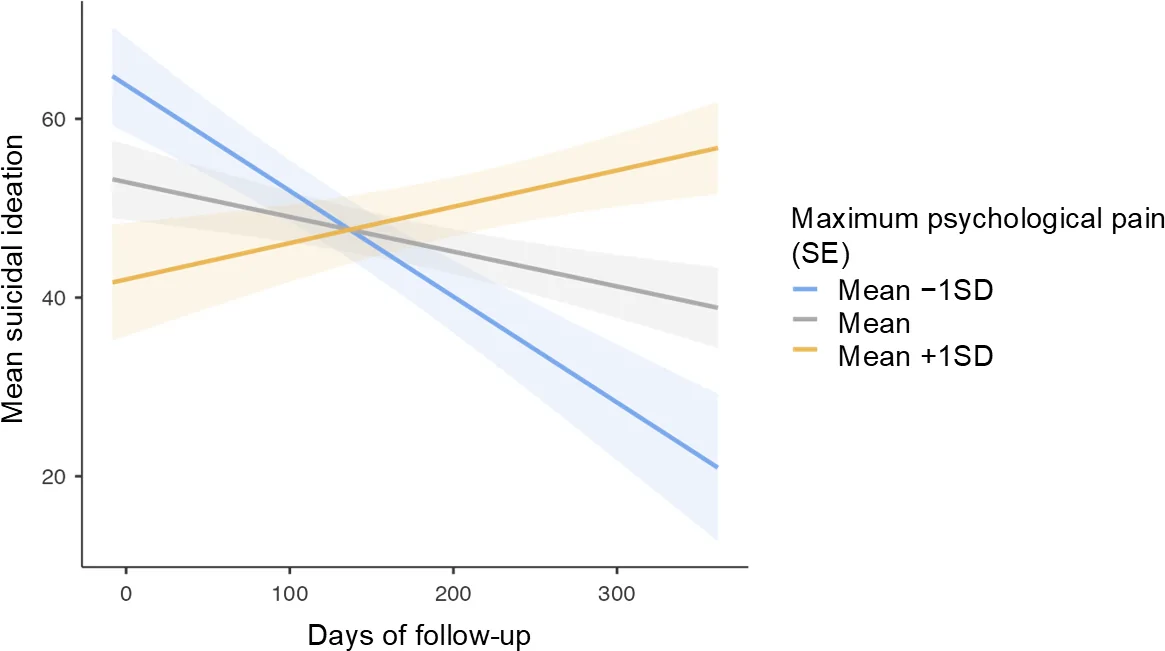
Association between baseline psychological pain and ecological momentary assessment–measured suicidal ideation. IDS-C: Inventory of Depressive Symptomatology–Clinician Rated.

Psychological pain itself did not predict intraindividual variability (*P=.*15) or the mean of SI (*P=.*16). The interaction psychological pain × days of follow-up predicted the intraindividual mean of SI (*R*^2^=0.34; β=0.024, SE 0.001; *t*_98_=3.95; *P*<.001). Simple slopes showed significant results for the –1SD psychological pain (β=−0.12, SE 0.030; *P*<.001) but not for the +1 SD of psychological pain (β=0.04, SE 0.023; *P*=.10), showing that patients with lower psychological pain and longer follow-up experience less SI (Figure 3). The interaction psychological pain*days of follow-up did not predict the intraindividual variability of SI (*P=.*08).

**Figure 4.**
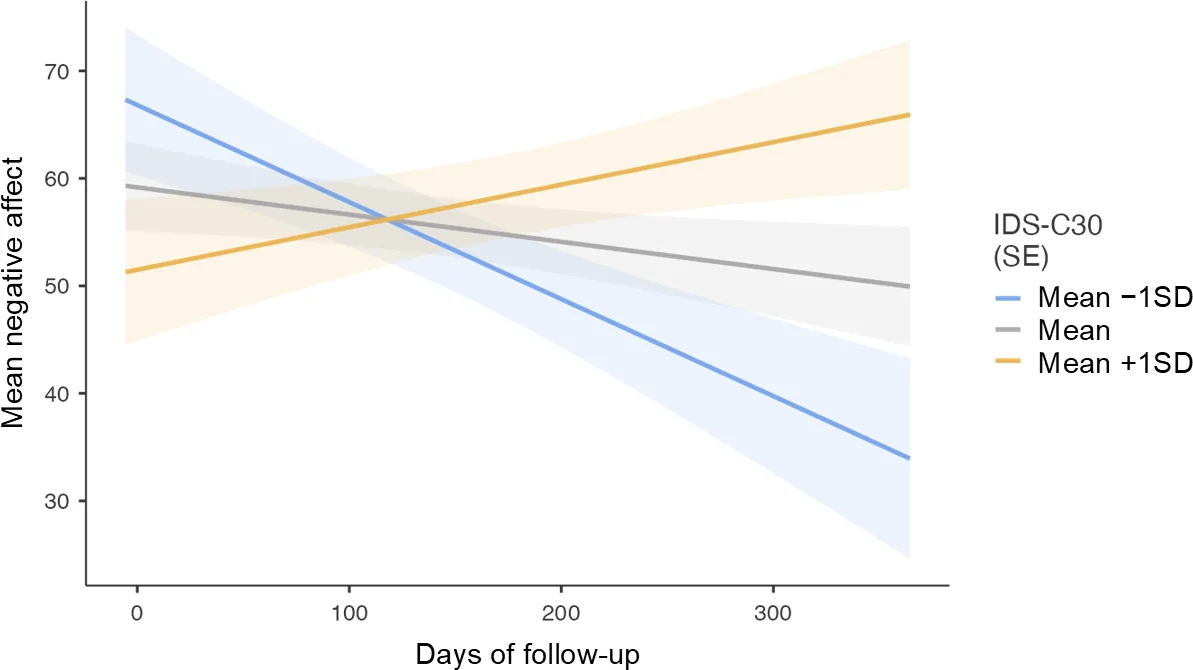
Association between baseline depression and ecological momentary assessment–measured negative affect. IDS-C: Inventory of Depressive Symptomatology—Clinician Rated.

Regarding depression, the effect of depression at baseline itself did not predict intraindividual variability (*P=.*75) or mean of negative affect (*P=.*11). The interaction depression*days of follow-up predicted the intraindividual mean of negative affect (*R*^2^=0.17; β=0.006, SE 0.003; *t*_98_=2.35; *P*=.03). Simple slopes showed significant trend results for the +1 SD of psychological pain (β=0.06, SE 0.03; *P*=.06) but not significant results for the –1 SD psychological pain (β=−0.06, SE 0.04; *P*=.18). Thus, patients with higher depression and longer follow-up experience greater negative affect (Figure 4). The interaction depression × days of follow-up did not predict the intraindividual variability of negative affect (*P=.*87).

Results for variability in SI (depression *P*=.69; depression × days of follow-up *P*=.99) or SI mean (depression *P*=.26; depression × days of follow-up *P*=.51) using depression as a predictor did not show statistically significant results.

## Discussion

### Summary of Results

This study examined SI and its associations with other psychological and behavioral variables in a sample of almost 100 high-risk outpatients who were monitored for 6-months. Trajectories (slopes) of SI, negative affect, interpersonal difficulties, sleep disturbances, and appetite issues did not demonstrate significant change over time. However, SI was significantly associated with negative affect and interpersonal difficulties. Additionally, substantial within-person variability in SI was observed. Notably, individuals who reported higher levels of psychological distress at the beginning of the study exhibited increased levels of negative affect and SI throughout the follow-up period.

### Fluctuations in EMA Variables Over Time

Considerable within-person variability was observed for all EMA-tracked variables. Relatively low ICCs suggest that these constructs reflect dynamic, state-like fluctuations rather than stable traits [32]. The pronounced variability in SI and its related factors emphasizes the fluctuating nature of suicide risk. Bernanke et al [33] have proposed two subtypes of SI: one that is reactive to stress and tends to spike following acute stressors, and another that is more chronic and associated with persistent depressive states. This distinction may partly account for the variability observed in our data, as individual emotional responses are likely shaped by personal experiences.

Consistent with this, Bonilla-Escribano et al [34] conducted an EMA study involving 275 psychiatric outpatients and nearly 50,000 responses. Their findings indicated that high SI variability was associated with greater instability in social withdrawal, sleep, and perceived social support.

The dynamic interplay between negative affect, interpersonal difficulties, and SI, as observed in our study, supports the need for personalized, real-time monitoring strategies [10]. This reinforces recent critiques of traditional clinical assessments, which often fail to capture moment-to-moment fluctuations in suicide risk [6]. Our results emphasize the value of EMA in capturing these rapid shifts, which standard tools may overlook.

### Associations Between EMA-Measured Variables

One of the key findings was the significant association between SI and negative affect, as well as interpersonal difficulties, as measured via EMA. These results are consistent with prior research identifying these variables as critical components in the development of suicidal thoughts. In particular, our findings align with existing literature linking SI to interpersonal distress, including feelings of loneliness and of being a burden to others [35,36]. Similarly, Bentley et al [37] identified emotions such as anxiety, shame, and self-hatred as predictors of subsequent SI using EMA. These results echo the core tenets of the Interpersonal Theory of Suicide, which posits that a lack of belonging and the perception of being a burden are fundamental drivers of SI [38].

However, sleep and appetite disturbances were not significantly associated with increased SI in this sample. This diverges from earlier studies conducted by our group [39], which focused specifically on passive SI. Differences in sample size and the more pronounced role of negative affect may explain these inconsistencies.

We also found that greater variability in negative affect, interpersonal experiences, sleep, and appetite within individuals corresponded with greater variability in SI. Whether such fluctuation in SI translates into an increased risk of suicide attempts remains an open question. However, evidence from studies such as that of Oquendo et al [40], which followed 51 patients with depression for two years, suggests that higher SI variability may increase reactivity to stressors and elevate the risk of impulsive suicidal behavior.

### Baseline Clinical Predictors of EMA-Measured Variables

Baseline psychological pain was found to have a significant influence on SI throughout the EMA follow-up. The relationship between psychological distress and suicidal thoughts can be explained by several theoretical models. For example, Shneidman [41] identified unbearable psychological pain as a key factor in suicidal behavior, while Baumeister [42] proposed that suicide may be an attempt to escape distress caused by perceived failure or unmet expectations. More recently, Klonsky and May [43] 3-step theory emphasized the role of psychological pain and hopelessness in the development of SI.

Regarding depression, we found that baseline severity was associated with increased negative affect over time. However, no significant association was found between depression and SI. Since negative affect includes components such as hopelessness, anxiety, sadness, anger, and restlessness—many of which overlap with depressive symptomatology—this association is expected. Nevertheless, our findings suggest that SI is a more multifaceted construct that is not exclusively linked to depressive disorders. This highlights the importance of considering other psychiatric conditions, particularly those characterized by emotional dysregulation, when assessing suicide risk.

### Strengths and Limitations

This study has several strengths. The 6-month follow-up period allows for the examination of longer-term patterns beyond acute suicidal crises. Participants were recruited from real-world mental health outpatient settings and represented a high-risk clinical population, enhancing ecological validity. EMA items were derived from validated instruments and administered using a rotating sampling strategy designed to improve feasibility and adherence over time. Finally, the use of mixed-effects models allowed for the analysis of repeated EMA measurements while accounting for individual differences in response patterns.

Despite these strengths, several limitations should be acknowledged. First, the observational design does not allow causal conclusions to be drawn about the associations between SI and EMA variables. Second, EMA items were administered using a rotating sampling strategy to reduce participant burden; however, this limited temporal continuity across constructs and prevented the examination of lagged relationships. In addition, although the EMA design aimed to improve feasibility and maximize long-term adherence [8,20], some features—particularly the variation in temporal framing of items (eg, momentary, since the last 24 h, in the past few days)—could be refined in future studies to improve alignment between the time frame, response scale, and expected construct variability. Furthermore, although EMA prompts were delivered at random times, participants were allowed to complete questionnaires until midnight, which may have introduced recall bias if responses did not reflect the moment of the prompt, or selection bias if participants reported on a different moment of their choosing. Third, baseline data on psychological pain and depression were only available for a subsample of 37 participants, reducing the statistical power of secondary analyses. Finally, response rates could not be calculated for individual EMA items due to the rotating and partially random sampling design. The overall response rate was relatively low, likely due to the extended follow-up period of our study. It is important to note that EMA studies are usually conducted over shorter periods, often lasting only a few weeks (8). Therefore, the longer duration of our study may have negatively affected long-term participant adherence and should be considered when interpreting the findings.

### Future Research Directions

Future studies should explore integrating EMA into routine clinical practice. One promising approach is the use of EMIs, which can deliver personalized, context-aware feedback based on momentary risk profiles. Furthermore, research should aim to identify subgroups of patients who may benefit from specific therapeutic strategies. For instance, individuals with heightened emotional variability may respond better to interventions based on emotion regulation, while those experiencing ongoing interpersonal difficulties could benefit from targeted social support programs [17].

### Conclusions

This study is one of the few to use smartphone-based EMA to monitor self-injury over an extended period [13]. EMA is a useful method for capturing short-term suicide risk factors. It is particularly important to consider negative emotions and interpersonal issues in suicide prevention. The high within-person variability observed in SI and its correlates emphasizes the dynamic and fluctuating nature of suicide risk. Moving forward, integrating EMA-based monitoring into clinical care could facilitate real-time, personalized interventions aimed at mitigating suicide risk in individuals at risk.

## Supplementary material

10.2196/85073 Multimedia Appendix 1 Ecological momentary assessment questionnaire.

## Abbreviations

EMA: ecological momentary assessment
EMI: ecological momentary intervention
ICC: intraclass correlation coefficient
IDS-C: Inventory of Depressive Symptomatology—Clinician Rated
RCT: randomized controlled trial
SI: suicidal ideation
STB: suicidal thoughts and behavior
YPLL: years of potential life lost
