# Supplementary material for: Association Between Suicidal Ideation and Negative Affect: 6-Month Ecological Momentary Assessment Study
Source: J Med Internet Res. 2026 Sep 23;28:e85073. doi: 10.2196/85073 (PMC13600663; doi:10.2196/85073)
Supplement: Supplementary file 1 [file jmir-v28-e85073-s001.docx]

| **Table 1.** Ecological Momentary Assessment (EMA) questionnaire. | | | | | | |
| --- | --- | --- | --- | --- | --- | --- |
| Area | Variable | Question | Minimum value | Maximum  value | Scoring | Source |
| **Suicidal Ideation (5 questions)** | Passive SI | My wish to live is | No wish to die | Maximum wish to die | 1-7 | SSPQ ^[26]^ |
|  | Active SI | Do you have thoughts that you would be better off dead or of hurting yourself in some way? | Not at all | Nearly every day | 0-3 | PHQ-9 ^[27]^ |
|  |  | How intense is your desire to kill yourself right now? | Absent/no desire | Extremely intense | 0-5 | Based on prior EMA studies ^[15,20]^ |
|  |  | How able are you to keep yourself safe right now? | I definitely can keep myself safe | I definitely cannot keep myself safe | 1-5 |  |
| **Non-suicidal self-injury (2 questions)** | Non-suicidal self-injury | At any point in the last 24 hours, did you harm yourself on purpose without the intention to die? | Yes | No | Yes/No | Based on prior EMA studies ^[17]^ |
|  |  | Since the last prompt, have you felt an urge or wanted to harm or injure yourself on purpose, without wanting to die? | Not at all | Extremely | 1-5 | Based on prior EMA studies ^[21]^ |
| **Affect (9 questions)** | Psychological pain | I feel psychological pain | No pain | Maximum pain | 1-7 | SSPQ ^[26]^ |
|  | Stress | I feel stressed out today (with pressure, overwhelmed) | No stress | Maximum stress | 1-7 |  |
|  | Restlessness | I feel restless (agitated), with the need to keep moving | No restlessness | Maximum restlessness | 1-7 |  |
|  | Hopelessness | I feel full of hope | No hope | Maximum hope | 1-7 |  |
|  | Anger towards self | I feel hatred or anger towards myself | No hatred | Maximum hatred | 1-7 |  |
|  | Anger towards others | I feel hatred or anger towards others | No hatred | Maximum hatred | 1-7 |  |
|  | Anxiety | At this moment I feel nervous | Very slightly or not at all | Extremely | 1-5 | PANAS ^[28]^ |
|  | Depression | At this moment I feel sad | Very slightly or not at all | Extremely | 1-5 |  |
|  | Happiness | At this moment I feel happy | Very slightly or not at all | Extremely | 1-5 |  |
| **Interpersonal experiences (11 questions)** | Thwarted belonginess | I wish there was a trusted person with whom I can talk about all my personal issues | Not at all | Absolutely | 1-7 | SSPQ ^[26]^ |
|  | Thwarted belonginess | I feel like an outsider | Not at all | Absolutely | 1-7 |  |
|  | Lack of recognition | I wish I received more recognition and love from others | Not at all | Absolutely | 1-7 |  |
|  | Lack of independence | I have the impression that important people around me want to decide for me what I should think and do | Not at all | Absolutely | 1-7 |  |
|  | Criticism | Since the last prompt have you felt insulted or criticized? | Not at all | Extremely | 1-5 | Based on prior EMA studies ^[21]^ |
|  | Thwarted belonginess | Since the last prompt have you felt rejected, abandoned, excluded, or left out? | Not at all | Extremely | 1-5 |  |
|  | Perceived burdensomeness | I believe I’m contributing to the well-being of my family/friends | Not at all | Absolutely | 1-7 | SSPQ ^[26]^ |
|  | Perceived burdensomeness | I believe I’m contributing to the well-being of the people around me | Not at all | Absolutely | 1-7 |  |
|  | Thwarted belonginess | I feel disconnected from other people | Not at all | Absolutely | 1-7 |  |
|  | Perceived burdensomeness | I feel like a burden to others | Not at all true for me | Very true for me | 1-7 | INQ ^[29]^ |
|  | Perceived burdensomeness | I feel useless | Not at all true for me | Very true for me | 1-7 |  |
| **Sleep (4 questions)** | Sleep maintenance | Last night I had trouble staying asleep | None | Very severe | 0-4 | SSPQ ^[26]^ |
|  | Sleep-derived quality of life | Others think that sleep problems affect my quality of life | Not at all | Absolutely | 1-7 |  |
|  | Sleep dissatisfaction | Today I am satisfied with my sleep | Very unsatisfied | Very satisfied | 1-7 |  |
|  | Sleep-derived interference with daily activity | My sleep problems are interfering with my daily activity | Not at all | Very much | 0-4 |  |
| **Eating (3 questions)** | Appetite | In the last few days, I have been hungry… | Never | All the time | 0-4 | SSPQ ^[26]^ |
|  | Taste | In the last days when I eat, the food tastes… | Very bad | Very good | 0-4 |  |
|  | Number of meals | In the last few days, I usually do… | Less than one meal a day | More than three meals a day | 0-4 |  |
| INQ=Interpersonal Needs Questionnaire; PANAS= Positive and Negative Affect Schedule; PHQ-9=Patient Health Questionnaire-9; SI=Suicidal Ideation; SSPQ=Salzburg Suicide Process Questionnaire. | | | | | | |

**References**

15. Parrish EM, Chalker SA, Cano M, Moore RC, Pinkham AE, Harvey PD, et al. Ecological momentary assessment of interpersonal theory of suicide constructs in people experiencing psychotic symptoms. J Psychiatr Res. 2021;140:496–503. [doi:10.1016/J.JPSYCHIRES.2021.06.022](https://doi.org/10.1016/J.JPSYCHIRES.2021.06.022)
17. Burke LE, Shiffman S, Music E, Styn MA, Kriska A, Smailagic A, et al. Ecological Momentary Assessment in Behavioral Research: Addressing Technological and Human Participant Challenges. J Med Internet Res. 2017;19(3):e77. [doi:10.2196/jmir.7138](https://doi.org/10.2196/jmir.7138).
20. Porras-Segovia A, Díaz-Oliván I, Barrigón, ML, Moreno M, Artés-Rodríguez A, Pérez-Rodríguez MM, et al. Real-world feasibility and acceptability of real-time suicide risk monitoring via smartphones: A 6-month follow-up cohort. J Psychiatr Res. 2022;149:145–154. [doi:10.1016/J.JPSYCHIRES.2022.02.026](https://doi.org/10.1016/J.JPSYCHIRES.2022.02.026)
21. Porras-Segovia A, De Granda-Beltrán AM, Gallardo C, Abascal-Peiró S, Barrigón ML, Artés-Rodríguez A, et al. Smartphone-based safety plan for suicidal crisis: The SmartCrisis 2.0 pilot study. J Psychiatr Res. 2024; 169:284–291. doi:10.1016/j.jpsychires.2023.11.039.
26. Fartacek C, Schiepek G, Kunrath S, Fartacek R, Plöderl M. Real-Time Monitoring of Non-linear Suicidal Dynamics: Methodology and a Demonstrative Case Report. Front Psychol. 2016;7:130. doi:10.3389/fpsyg.2016.00130.
27. Kroenke K, Spitzer RL, Williams JB. The PHQ-9: validity of a brief depression severity measure. J Gen Intern. Med 2001;16:606–13. doi: 10.1046/j.1525-1497.2001.016009606.x.
28. Watson D, Clark LA, Tellegen A. Development and validation of brief measures of positive and negative affect: the PANAS scales. J Pers Soc Psychol. 1988;54:1063–70. doi: 10.1037//0022-3514.54.6.1063.
29. Van Orden KA, Cukrowicz KC, Witte TK, Joiner TE. Thwarted belongingness and perceived burdensomeness: construct validity and psychometric properties of the interpersonal needs questionnaire. Psychol Assess. 2012;24:197–215. doi: 10.1037/a0025358.
